# Treatment of oral lichen planus using deucravacitinib

**DOI:** 10.1016/j.jdcr.2023.12.006

**Published:** 2023-12-27

**Authors:** Mindy Vu, Rama Abdin, Naiem T. Issa

**Affiliations:** a Forefront Dermatology, Vienna, Virginia; b Issa Research and Consulting, LLC, Springfield, Virginia; c Charles E. Schmidt College of Medicine, Florida Atlantic University, Boca Raton, Florida; d Dr. Phillip Frost Department of Dermatology and Cutaneous Surgery, University of Miami Miller School of Medicine, Miami, Florida

**Keywords:** deucravacitinib, oral lichen planus, treatment, tyrosine kinase 2 inhibitor

## Introduction

Oral lichen planus (OLP) is an immune-mediated, lichenoid inflammatory condition involving the oral mucosa. OLP is classically described as bilateral, reticular white lesions in the oral mucosa; however, the disease displays a range of morphologies including erosions, plaques, white papules, white plaques, and/or blisters, which may be unilateral or bilateral.^1^^,^^2^ Atrophic lesions may be painful because of a loss of the mucosal barrier and resultant increased sensitivity to strong flavors or acidic foods.^1^ The pathogenesis of OLP is driven by CD8^+^ and CD4^+^ T cells, which cause keratinocyte apoptosis and basement membrane disruption; however, this process is highly complex and not completely understood. Briefly, it begins with antigen-presenting cell stimulation and subsequent antigen-specific T-cell activation.^1^ T cells can be activated by antigen-presenting cells at lesional sites or at distal lymph nodes.^1^ These activated T cells migrate to OLP lesional sites through chemokine and adhesion molecule signaling.^1^ The progression of OLP pathogenesis and chronicity is fostered by mast cell and macrophage degranulation, which releases signaling molecules that promote T-cell recruitment to, and migration within, the lesional skin.^1^ Proinflammatory cytokines involved in this process include, but are not limited to, tumor necrosis factor-alfa, interferon gamma, interleukin (IL)-1β, IL-12, and IL-6.^1^ This intricate disease process involves other pathogenic arms, of which this report will focus on the role of the IL-17/IL-23 axis.

The current standard treatment for OLP includes the use of topical corticosteroids.^2^ Because of the location of lesions in the oral cavity, effective localized treatment is difficult. In recalcitrant cases, treatment with systemic steroids may be pursued. The extensive short- and long-term side effects of corticosteroid use may limit patient use and tolerability.

Recently, IL-17 and IL-23 signaling pathways have been implicated in OLP.^3^ IL-17 is thought to enhance T-cell governed reactions and upregulate key chemokines and cytokines involved in tissue remodeling and proinflammatory pathways.^2^^,^^4^ Recent evidence has supported a prominent role of IL-17 and IL-23 in the pathogenesis of lichen planus through the successful treatment of the disease with anti-IL-17 and -IL-23 agents.^2^^,^^5^ Two patients with advanced mucocutaneous lichen planus reported clinical improvement with secukinumab, an anti-IL-17A antibody, and demonstrated a decrease in T-cell infiltrates within lesions.^5^ A reduction in serum levels of IL-17A^+^ T cells was also demonstrated in patients treated with ustekinumab, an anti-IL-12/IL-23 biologic.^5^ Given the emerging role of the IL-17/IL-23 axis in OLP and the success of IL-17/IL-23-targeting antibodies, we hypothesized that targeting tyrosine kinase 2 (TYK2) may also be beneficial. TYK2 is a Janus kinase (JAK) protein that relays intracellular signaling from the binding of IL-23 to its membrane receptor on T lymphocytes, thereby leading to the production of IL-17.^6^ Selective inhibition of TYK2 by deucravacitinib has therefore been effective in psoriasis.^6^ Herein, we describe a case of long-standing treatment recalcitrant OLP in an adult woman who responded positively to treatment with deucravacitinib 6 mg daily for 5 months.

## Case study

**Fig 1:**
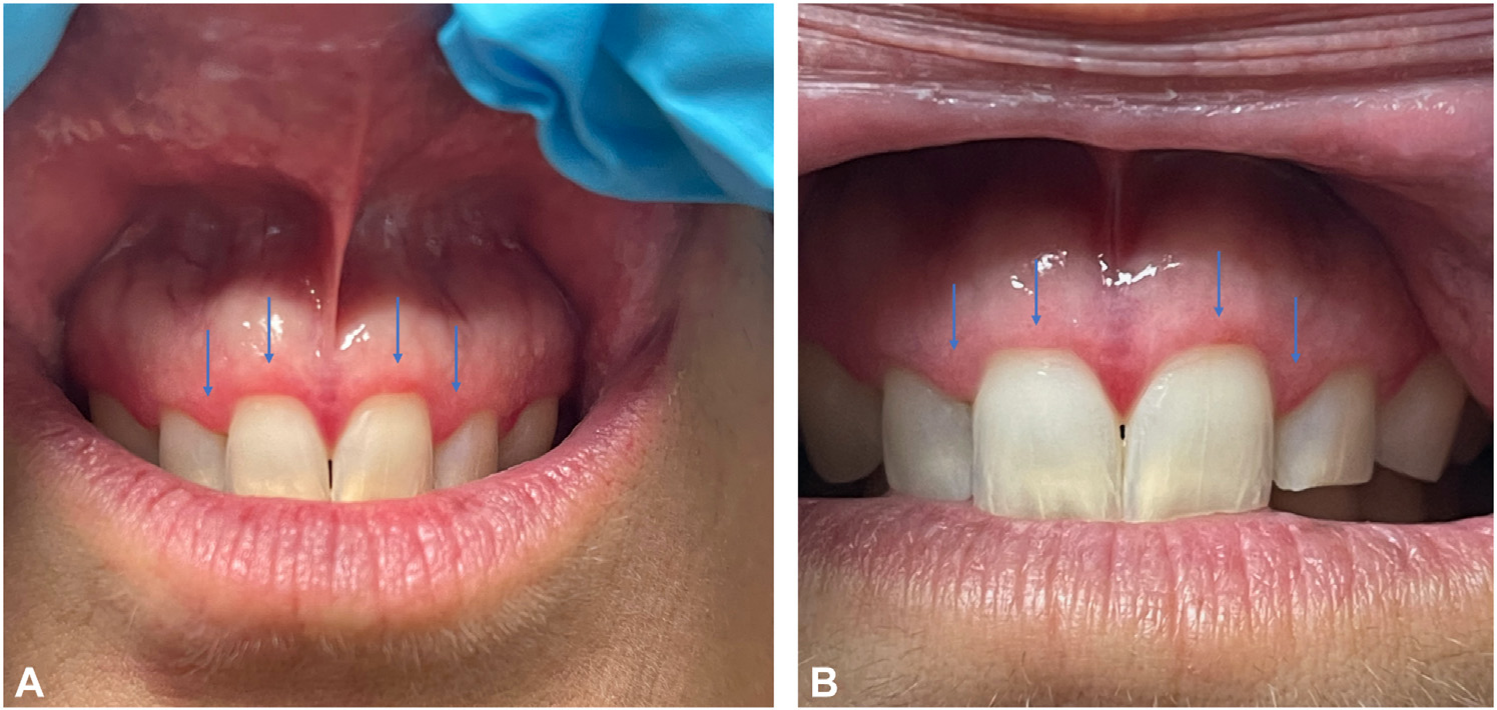
Oral lichen planus **(A)** before treatment and **(B)** after treatment with deucravacitinib over 5 months.

**Fig 2:**
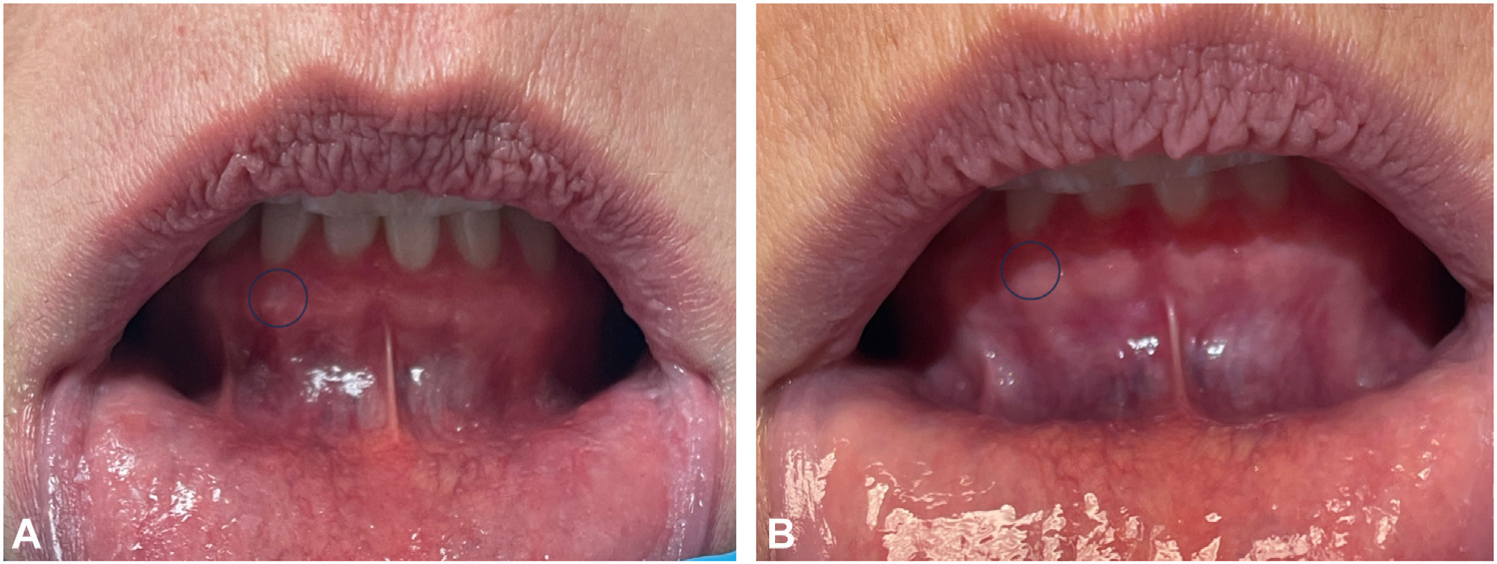
Oral lichen planus **(A)** before treatment and **(B)** after treatment with deucravacitinib over 5 months.

A 52-year-old Caucasian woman presented with an approximately 12-year history of irregular, tender, maxillary, mandibular, and buccal mucosal lesions associated with dysphagia and sensitivity to acidic foods (Figs 1, *A*; 2, *A*; and 3, *A*). Treatment before presentation included topical steroids, steroid rinses, oral prednisone, and topical tacrolimus, with no relief. During the course of her disease, the patient underwent multiple biopsies of the buccal mucosa, which initially revealed atypical epithelial cells suspicious for squamous cell carcinoma, treated with carbon dioxide (CO_2_) resurfacing. Despite these therapies, the patient continued to experience worsening symptoms. Diagnosis of OLP was then confirmed using a repeat biopsy of the lesional buccal mucosa.

**Fig 3:**
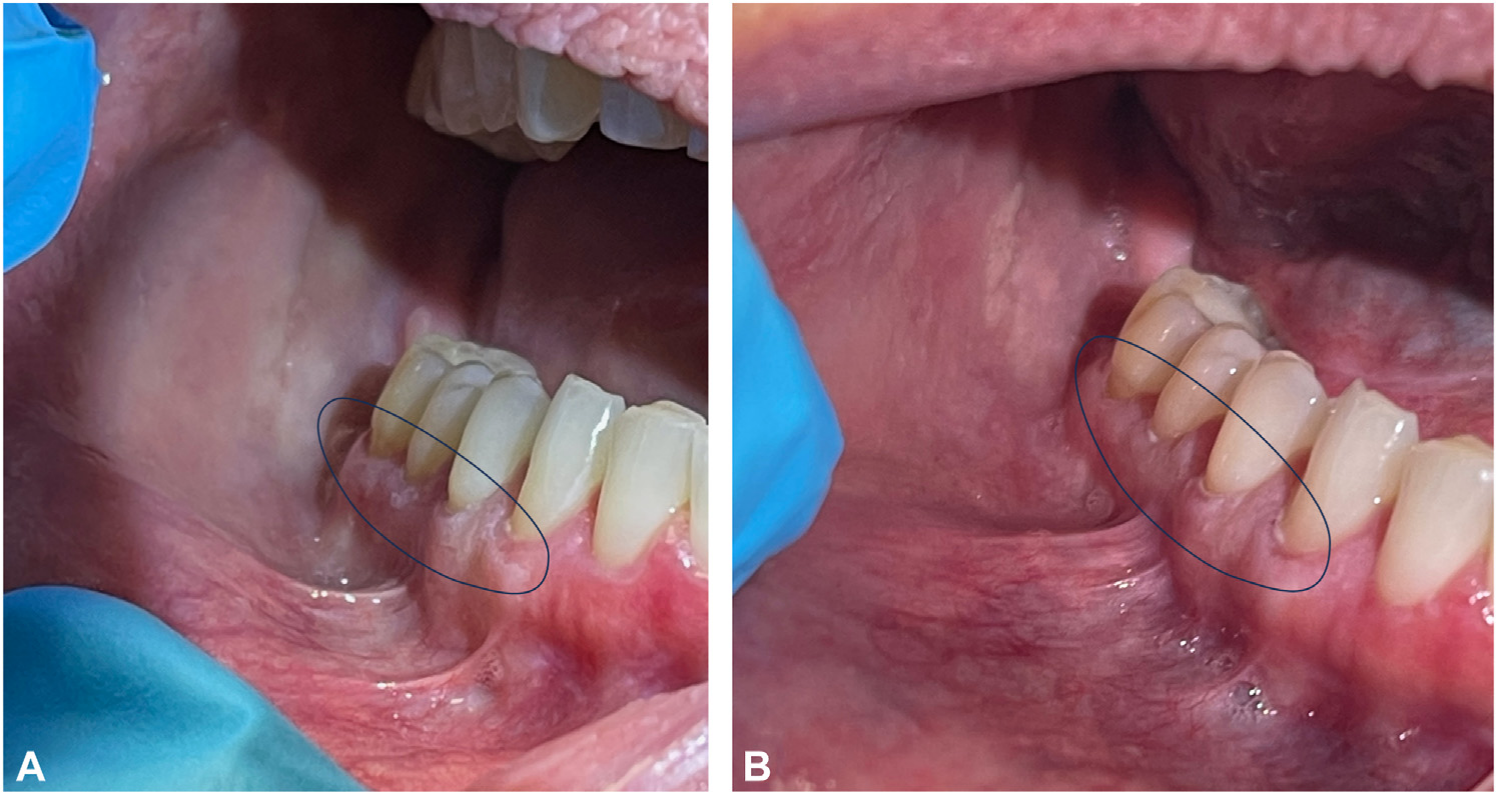
Oral lichen planus **(A)** before treatment and **(B)** after treatment with deucravacitinib over 5 months.

Upon presentation, patient was treated with deucravacitinib 6 mg tablets daily and endorsed significant improvement in pain as well as reduction of erythema and ulcerations within 5 months (Figs 1, *B*; 2, *B*; and 3, *B*). The patient also reported perioral acneiform lesions 1 month after initiation of deucravacitinib, which self-resolved approximately 1 to 2 weeks later without intervention. The patient remained symptom free at her 6-month follow-up appointment.

## Discussion

Deucravacitinib is a novel selective allosteric inhibitor of TYK2 currently approved for the treatment of plaque psoriasis and is currently under investigation for psoriatic arthritis.^6^ TYK2 is a member of the JAK family that promotes inflammation and autoimmune responses through JAK-STAT signaling.^6^ Given the role of interferon gamma and other proinflammatory cytokines in the pathogenesis of lichen planus and the ability of JAK inhibitors to blunt this response, the use of these agents has been reported to treat lichen planus.^7^ In fact, use of both baricitinib and upadacitinib has been described in case reports to treat OLP.^7^ Within the JAK family, TYK2 specifically mediates intracellular signaling of IL-23 via recruitment of STAT3 and ultimately transcription of IL-17. Deucravacitinib has been shown to decrease IL-17 and IL-23 as well as inhibit T helper 17 cell development.^6^ Given the critical role of the IL-17/IL-23 axis and T helper 17 cells in numerous cutaneous and oral mucosal inflammatory conditions, we hypothesized that deucravacitinib should also be effective for OLP.^2^^,^^8^, ^9^, ^10^

To our knowledge, the case presented here is notable because it is the first reported use of TYK2 inhibition and deucravacitinib for the successful treatment of OLP. Our patient exhibited long-standing OLP that was resistant to standard treatments that included prolonged use of topical and oral corticosteroids along with topical tacrolimus. Despite resistance to these treatments, she responded to oral deucravacitinib. Furthermore, although the effect of deucravacitinib in this case is relatively slow (5 months), this is in line with other observations where IL-17 and IL-23 biologics have been successfully used for treating OLP.^2^

## Conclusion

OLP is a chronic and painful immune-mediated condition with limited therapeutic options. To our knowledge, our findings are the first to show that TYK2 inhibition with deucravacitinib is a novel and safe treatment option for OLP. The utility of deucravacitinib in this case further implicates the IL-17/IL-23 axis in OLP. Given its safety profile and rare mechanism of action as an allosteric and targeted TYK2 inhibitor, deucravacitinib should be further investigated for OLP as well as cutaneous lichen planus and lichen planopilaris in future larger scale clinical trials.

## Conflicts of interest

None disclosed.
